# Cost–effectiveness of screening for active cases of tuberculosis in Flanders, Belgium

**DOI:** 10.2471/BLT.16.169383

**Published:** 2016-11-03

**Authors:** G Suzanne A Smit, Ludwig Apers, Wouter Arrazola de Onate, Philippe Beutels, Pierre Dorny, An-Marie Forier, Kristien Janssens, Jean Macq, Ruud Mak, Sandrina Schol, Dirk Wildemeersch, Niko Speybroeck, Brecht Devleesschauwer

**Affiliations:** a Department of Virology, Parasitology and Immunology, Ghent University, Ghent, Belgium.; b Institute of Tropical Medicine, Antwerp, Belgium.; c Flemish Association for Respiratory Health and Tuberculosis Control, Brussels, Belgium.; d Centre for Health Economics Research & Modelling Infectious Diseases, University of Antwerp, Antwerp, Belgium.; e Agency for Care and Health, Government of Flanders, Brussels, Belgium.; f Institute of Health and Society, Université catholique de Louvain, Brussels, Belgium.; g Scientific Institute of Public Health (WIV-ISP), Department of Public Health and Surveillance, Rue Juliette Wytsmanstraat 14, 1050 Brussels, Belgium.

## Abstract

**Objective:**

To assess the cost–effectiveness of the tuberculosis screening activities currently funded by the Flemish government in Flanders, Belgium.

**Methods:**

After estimating the expenses for 2013–2014 of each of nine screening components – which include high-risk groups, contacts and people who are seeking tuberculosis consultation at a centre for respiratory health care – and the associated costs per active case of tuberculosis identified between 2007 and 2014, we compared the cost–effectiveness of each component. The applied perspective was that of the Flemish government.

**Findings:**

The three most cost-effective activities appeared to be the follow-up of asylum seekers who were found to have abnormal X-rays in initial screening at the Immigration Office, systematic screening in prisons and contact investigation. The mean costs of these activities were 5564 (95% uncertainty interval, UI: 3791–8160), 11 603 (95% UI: 9010–14 909) and 13 941 (95% UI: 10 723–18 201) euros (€) per detected active case, respectively. The periodic or supplementary initial screening of asylum seekers and the screening of new immigrants from high-incidence countries – which had corresponding costs of €51 813 (95% UI: 34 855–76 847), €126 236 (95% UI: 41 984–347 822) and €418 359 (95% UI: 74 975–1 686 588) – appeared much less cost-effective. Between 2007 and 2014, no active tuberculosis cases were detected during screening in the juvenile detention centres.

**Conclusion:**

In Flanders, tuberculosis screening in juvenile detention centres and among new immigrants and the periodic or supplementary initial screening of asylum seekers appear to be relatively expensive ways of detecting people with active tuberculosis.

## Résumé

**Objectif:**

Évaluer la rentabilité des activités de dépistage de la tuberculose actuellement financées par le gouvernement flamand en Flandre, en Belgique.

**Méthodes:**

Après avoir estimé les dépenses pour 2013–2014 au titre de chacun des neuf composants du dépistage – notamment les groupes à haut risque, les contacts et les personnes venant en consultation pour la tuberculose dans un centre de traitement des problèmes respiratoires – ainsi que les coûts associés par cas de tuberculose active identifié entre 2007 et 2014, nous avons comparé la rentabilité de chaque composant. La perspective appliquée était celle du gouvernement flamand.

**Résultats:**

Il est apparu que les trois activités les plus rentables étaient le suivi des demandeurs d'asile dont les radiographies réalisées lors du dépistage initial à l'Office des étrangers étaient anormales, le dépistage systématique dans les prisons et la recherche des contacts. Le coût moyen de ces activités était respectivement de 5564 (intervalle d'incertitude de 95%, II: 3791–8160), 11 603 (II 95%: 9010–14 909) et 13 941 (II 95%: 10 723–18 201) euros (€) par cas actif détecté. Le dépistage initial périodique ou complémentaire des demandeurs d'asile et le dépistage des nouveaux immigrants originaires de pays à forte incidence – dont le coût était respectivement de 51 813 € (II 95%: 34 855–76 847), 126 236 € (II 95%: 41 984–347 822) et 418 359 € (II 95%: 74 975–1 686 588) – se sont révélés beaucoup moins rentables. Entre 2007 et 2014, aucun cas de tuberculose active n'a été détecté lors du dépistage dans les centres de détention pour mineurs.

**Conclusion:**

En Flandre, le dépistage de la tuberculose dans les centres de détention pour mineurs et chez les nouveaux immigrants ainsi que le dépistage initial, périodique ou complémentaire, des demandeurs d'asile, est un moyen relativement coûteux de détecter les cas de tuberculose active.

## Resumen

**Objetivo:**

Evaluar la rentabilidad de las actividades de detección de tuberculosis actualmente financiadas por el gobierno flamenco en Flandes, Bélgica.

**Métodos:**

Tras calcular los gastos de 2013–2014 de cada uno de los nueve componentes de los exámenes de detección (que incluyen grupos de alto riesgo, contactos y personas que buscan consulta para la tuberculosis en un centro de atención respiratoria) y los costes asociados por caso activo de tuberculosis identificados entre 2007 y 2014, se comparó la rentabilidad de cada componente. Se aplicó la perspectiva del gobierno flamenco.

**Resultados:**

Parece que las tres actividades más rentables fueron el seguimiento de los buscadores de asilo con unas radiografías poco habituales en el examen de detección inicial en la Oficina de Inmigración, los exámenes de detección sistemáticos en prisiones y la investigación de contactos. Los costes medios de estas actividades fueron de 5 564 (intervalo de incertidumbre, II, del 95%: 3 791–8 160), 11 603 (II del 95%: 9 010–14 909) y 13 941 (II del 95%: 10 723–18 201) euros (€) por caso activo detectado respectivamente. Los exámenes de detección inicial periódicos o complementarios de los buscadores de asilo y los exámenes de detección de los nuevos inmigrantes de países con una alta incidencia (con costes correspondientes de 51 813 (II del 95%: 34 855–76 847), 126 236 (II del 95%: 41 984–347 822) y 418 359 (II del 95%: 74 975–1 686 588) euros) parecieron ser mucho menos rentables. Entre 2007 y 2014 no se detectaron casos de tuberculosis activa durante los exámenes de detección en centros penitenciarios juveniles.

**Conclusión:**

En Flandes, los exámenes de detección de tuberculosis en centros penitenciarios juveniles y aquellos realizados entre nuevos inmigrantes, así como los exámenes de detección inicial periódicos o complementarios de los buscadores de asilo, parecen ser formas relativamente caras de detectar a personas con tuberculosis activa.

## ملخص

**الغرض:**

تقييم فعالية تكلفة أنشطة فحص السل المُمولة حاليًا من قبل الحكومة الفلمنكية في فلاندرز ببلجيكا.

**الطريقة:**

بعد تقدير نفقات عامي 2013 و2014 لكلٍ من عناصر الفحص التسعة – والتي تشمل المجموعات المعرضة للخطر، والاتصالات، والأشخاص الذين يسعون لمشورة تتعلق بالسل في أحد مراكز الرعاية الصحية للجهاز التنفسي – والتكاليف المرتبطة لكل حالة نشطة من السل تم تحديدها بين عامي 2007 و2014، فقد قارنا فعالية التكلفة لكل مكون. وكان المنظور المطبق هو منظور الحكومة الفلمنكية.

**النتائج:**

اتضح أن الأنشطة الثلاثة الأكثر فعالية من حيث التكلفة هي متابعة طالبي اللجوء الذين خرجت صور الأشعة السينية التي تم إجراؤها لهم بنتائج غير طبيعية في الفحص الأولي في مكتب الهجرة، والفحص المنهجي في السجون، والتحري من خلال الاتصالات. وبلغ متوسط التكاليف لهذه الأنشطة 5564 (بهامش الشك بنسبة 95‏‏%، ويتراوح ما بين 3791-8160)، و11603 (بهامش الشك بنسبة 95‏%، ويتراوح ما بين 9010-149090) و13,941 (بهامش شك بنسبة 95‏‏%، ويتراوح ما بين 10723-18201) يورو للكشف عن كل حالة نشطة، على التوالي. بدا كلاً من الفحص الدوري أو الإضافي الأولي لطالبي اللجوء وفحص المهاجرين الجدد من الدول ذات نسب إصابة العالية - الذي ترتب عنه تكاليف تساوي 51813 يورو (بهامش شك بنسبة ‏95٪، يتراوح ما بين 34855–76847)، و126236 (بهامش الشك بنسبة 95‏%، ويتراوح ما بين 41984-347822)، و418359 (بهامش شك بنسبة 95‏%، يتراوح ما بين 74975 – 1686588) – أقل من حيث فعالية التكلفة. وبين عامي 2007 و2014، لم يتم الكشف عن أي حالات سل نشطة أثناء الفحص في مراكز احتجاز الأحداث.

**الاستنتاج:**

يبدو أن فحص السل في مراكز احتجاز الأحداث وبين المهاجرين الجدد والفحص الأولي الدوري أو التكميلي لطالبي اللجوء في فلاندرز يمثل طريقة مكلفة نسبيًا للكشف عن المصابين بحالات من مرض السل النشط.

## 摘要

**目的:**

旨在评估当前由比利时弗兰德斯佛兰德政府赞助的肺结核筛查活动的成本效益。

**方法:**

我们评估了 2013–2014 年 9 个筛查项目中每个项目的费用，这 9 个项目包括高危人群、接触人群及在呼吸科医疗保健中心寻求肺结核疾病咨询的人员。同时评估了 2007 到 2014 年之间确认的每例活动性肺结核病例的相关成本，随后，我们对各个组成部分的成本效益进行了比较。 应用前景与佛兰德政府相关。

**结果:**

三个成本效益最高的活动是对寻求庇护者的随访，这些寻求庇护者在移民局、监狱系统筛查和联系调查的初步筛查中表现出 X 射线异常的情况。 这些活动的均值成本为每个检测到的活动性病例 5564 欧元（95% 不确定区间，UI： 3791–8160），11 603 (95% UI: 9010–14 909) 和 13 941 (95% UI: 10 723–18 201)。 对寻求庇护者的定期或补充初步筛查以及对来自高发病国家的新移民筛查——相应的成本为 51 813 欧元 (95% UI: 34 855–76 847)，126 236 欧元 (95% UI: 41 984–347 822) 以及 418 359 欧元 (95% UI: 74 975–1 686 588) – 成本效益更低。 2007 到 2014 年间，在青少年拘留中心的筛查中，未发现活动性肺结核病例。

**结论:**

在弗兰德斯，青少年拘留中心和在新移民中筛查肺结核病例以及对寻求庇护者进行定期或补充初步筛查是一种相对昂贵的活动性肺结核人群筛查方式。

## Резюме

**Цель:**

Дать оценку экономической эффективности мероприятий скринингового обследования на туберкулез, финансируемых в настоящее время фламандским правительством во Фландрии, Бельгия.

**Методы:**

После подсчета расходов за период с 2013 по 2014 год каждого из девяти компонентов скринингового обследования, к которым относятся группы высокого риска, контакты и люди, стремящиеся получить консультацию по туберкулезу в центре лечения респираторных заболеваний, и связанных затрат для каждого случая активного туберкулеза, идентифицированного в период с 2007 по 2014 год, авторы сравнили экономическую эффективность каждого компонента. Оценка была проведена с позиций фламандского правительства.

**Результаты:**

В тройку наиболее экономически эффективных мероприятий вошли последующее врачебное наблюдение просителей убежища, у которых были обнаружены патологии в ходе рентгеновского обследования при первичном скрининговом обследовании в Иммиграционном бюро, систематическое скрининговое обследование в тюрьмах и отслеживание контактов. Средняя стоимость этих мероприятий составила 5564 евро (95%-й интервал неопределенности, ИН: 3791–8160), 11 603 евро (95%-й ИН: 9010–14 909) и 13 941 евро (95%-й ИН: 10 723–18 201) за каждый случай диагностированного активного заболевания соответственно. Периодическое или дополнительное первичное скрининговое обследование просителей убежища и скрининговое обследование новых иммигрантов из стран с высокой заболеваемостью, стоимость которых составляла соответственно 51 813 евро (95%-й ИН: 34 855–76 847), 126 236 евро (95%-й ИН: 41 984–347 822) и 418 359 евро (95%-й ИН: 74 975–1 686 588), оказались гораздо менее экономически эффективными. В период между 2007 и 2014 годами случаи активного туберкулеза во время скринингового обследования в изоляторах временного содержания несовершеннолетних правонарушителей обнаружены не были.

**Вывод:**

Во Фландрии скрининговое обследование на туберкулез в изоляторах временного содержания несовершеннолетних правонарушителей и периодическое или дополнительное первичное скрининговое обследование просителей убежища являются сравнительно дорогими способами выявления людей с активным туберкулезом.

## Introduction

In 2014, the global incidence of tuberculosis was 133 cases per 100 000 population.^1^ The World Health Organization (WHO) wishes to reduce the global incidence of tuberculosis below 10 cases per 100 000 population by 2035.^2^ Although the incidence in many high-income countries is already below this target level, tuberculosis remains a public health challenge.^3^ In most low-incidence countries there are low rates of tuberculosis transmission among the native population, a developing predominance of cases among the elderly – and other vulnerable and hard-to-reach risk groups – and individuals who carry latent tuberculosis infection for years before they become symptomatic.^4^^,^^5^

Low burdens of tuberculosis often lead to diminishing public awareness, inadequate political commitment, limited clinical and diagnostic expertise and the underfunding of tuberculosis research.^4^^,^^6^ At the same time, economic crises, growing inequity, increases in the incidence of diseases that are risk factors for tuberculosis and the dissemination of drug-resistant forms of *Mycobacterium tuberculosis* may all be contributing to an expansion in the burden posed by tuberculosis.^6^^–^^9^

If WHO’s tuberculosis goal for 2035 is to be achieved, global funding and commitment for tuberculosis care, control and prevention need to be increased^10^ and the cost-effective use of the resources that are – or become – available has to be optimized.^11^

WHO’s framework towards tuberculosis elimination in low-incidence countries, provides further goals and a customized strategy for countries such as Belgium.^6^^,^^12^^,^^13^ According to this framework, interventions and target groups must be prioritized based on an assessment – of the epidemiology of tuberculosis and health-care context in each setting – that is guided by data analysis and operational research.^6^ We therefore evaluated the cost–effectiveness of the tuberculosis screening activities, in northern Belgium, that are currently organized and funded by the Flemish government. The reporting of this study conforms to the Consolidated Health Economic Evaluation Reporting Standards (available from the corresponding author).^14^

## Methods

### Study setting

Flanders is the Dutch-speaking northern part of Belgium and has about 6.4 million inhabitants.^15^ The Flemish government’s Agency for Care and Health is responsible for the general coordination of the tuberculosis policy in Flanders. It also coordinates and partially carries out – and has the final responsibility for – the investigation of contacts of individuals with infectious tuberculosis. Because of resource constraints, a management contract was established between the Flemish government and the Flemish Association for Respiratory Health and Tuberculosis Control. The association has its headquarters in Brussels and eight regional centres for respiratory health care in Flanders. On behalf of the Flemish government, the association carries out active detection through contact investigation, systematic screening of five high-risk groups and the screening of other individuals – hereafter simply called others – who, though they do not belong to any specific risk groups, have presented for a consultation at a centre for respiratory health care.

The high-risk groups that are screened systematically but voluntarily by the association are: (i) all asylum seekers assigned to Flanders; (ii) all undocumented migrants i.e. individuals lacking legal status in Belgium – held in detention centres in Flanders; (iii) other immigrants who intend to stay in Flanders for more than three months and come from high-incidence countries – i.e. countries that have more than 50 active cases of tuberculosis per 100 000 population; (iv) prisoners in Flanders; and (v) the youth held in juvenile detention centres in Flanders. There is no legal obligation for screening – people are invited and strongly motivated but can refuse to participate. The association’s screening of asylum seekers consists of three components: (i) the follow-up of asylum seekers found to have an abnormal chest X-ray on initial screening by the Federal Agency for Asylum at the Immigration Office^16^ – hereafter called the follow-up of asylum seekers; (ii) supplementary initial screening – i.e. the screening of asylum seekers who have not been investigated by X-ray; and (iii) periodic screening, six and 12 months after arrival, of those not known to have active tuberculosis previously. Supplementary initial screening covers asylum seekers who are not eligible for an X-ray – e.g. children younger than five years, the physically disabled and pregnant women – and those who are not screened at the Immigration Office for other reasons.

In prisons that have their own X-ray facilities, the Justice Federal Public Service conducts the systematic screening of prisoners. In other prisons, the association conducts the screening, using a mobile X-ray unit. However, almost all X-rays of prisoners in Flanders are read by the association’s pulmonologists.

Compared with migrants in detention centres or prisoners, other new immigrants are relatively hard to reach because they are very diverse in terms of the languages they speak and rapidly disperse across Flanders. The association develops leaflets for documented immigrants, inviting them for screening.

The main goal of tuberculosis screening in Flanders is the detection of active infectious pulmonary tuberculosis – for which a chest X-ray represents the method of choice. However, for contact investigation and when investigating people who are not eligible for an X-ray, initial screening is based on a tuberculin skin test. If eligible for an X-ray, any contact found skin-test positive is subsequently investigated using a chest X-ray. For further diagnosis and treatment, suspected tuberculosis patients are referred to the curative sector.

The algorithms for the screening and treatment options that are provided in Belgium – from the perspective of the high-risk groups, contacts of a confirmed case and others are available from the corresponding author.

We only looked at the effectiveness of investments in tuberculosis control made by the Flemish government and therefore only conducted our study from the perspective of the Agency for Care and Health and the Flemish Association for Respiratory Health and Tuberculosis Control. We ignored the costs of the coordination and implementation of screening by the Federal Agency for Asylum and the Justice Federal Public Service, the costs of diagnosis and treatment in the curative sector, the costs of tuberculosis screening within programmes of occupational health care and within hospitals and any indirect costs for tuberculosis patients – e.g. income lost and travel expenses during their illness.

### Estimated numbers

We assessed the number of people screened and the number of active tuberculosis cases identified under the current tuberculosis policy. To increase the reliability of our estimates, we included all cases detected in the period 2007–2014. However, for contact investigation and follow-up of asylum seekers, only data from 2013 and 2014 were available.

### Costs

We investigated the screening-related expenditure of the Agency for Care and Health and the Flemish Association for Respiratory Health and Tuberculosis Control for the years 2013 and 2014. We used expert opinion to allocate fixed costs – e.g. overheads, rents, office supplies, staff, and organizational development – for each component of the screening policy. Allocation weights were based on the percentage of time each employee working on the tuberculosis programme spent on each component. These percentages were reported by the employees and recorded, anonymously, on questionnaires.

### Cost–effectiveness

Cost–effectiveness was evaluated over a one-year time horizon. With a policy of non-intervention as the comparator, the incremental cost–effectiveness ratio for each implemented component of the screening policy was defined as the ratio of the annual cost, in euros (€), over the period 2013–2014, to the annual number of active tuberculosis cases detected in the period 2007–2014. Using such ratios, we compared the cost–effectiveness of each component. As we used mean annual costs and outcomes, no discounting was necessary.

### Statistical analyses

Statistical uncertainty about the number of detected tuberculosis cases and the costs was investigated using Dirichlet distributions and 10 000 Monte Carlo simulations.^17^ We report the resulting means and 95% uncertainty intervals (UI). Data were collated into Excel 2010 (Microsoft, Redmond, United States of America) databases and analysed in R version 3.2.0.^18^

## Results

Most (336; 82%) of the 410 people with active tuberculosis reported in Flanders in 2014 were detected passively, when they sought health care. The other 74 people with active tuberculosis were detected during the screening of risk groups (28), contact investigation (34), the screening of others (3) or by unknown means (9).^12^ Among the reported cases, 29 had been born in eastern Europe and 208 outside Europe.

**Fig. 1:**
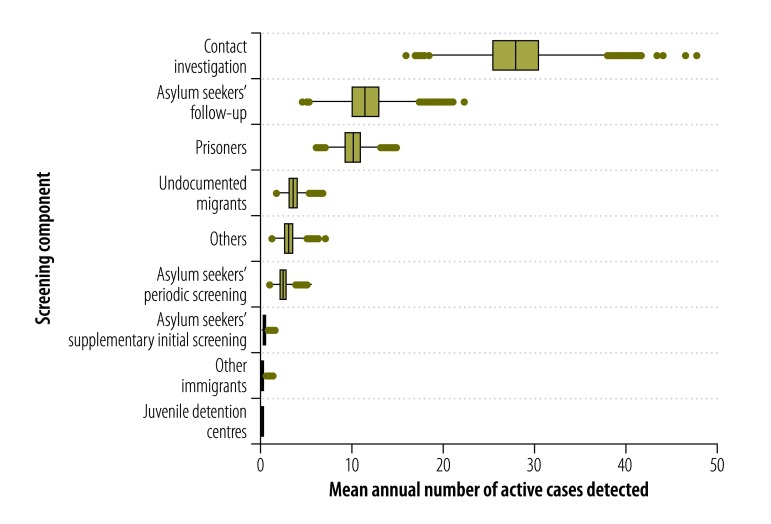
**Mean annual number of cases identified via each of nine components of the screening for active cases of tuberculosis, Flanders, Belgium, 2007–2014**

Notes: Others include individuals who do not belong to any specific risk groups but presented for consultation at a centre for respiratory health care. The boxplots reflect the statistical uncertainty in the mean annual number of detected cases. In each boxplot, the vertical line represents the median of all simulated values, the box indicates the first and third quartiles, the whiskers extend to 1.5 times the interquartile range, and the points represent simulated values outside that interval.

Table 1 summarizes the numbers of people screened in 2014 via each component of the screening policy, the corresponding numbers of active tuberculosis cases detected and the related costs. Fig. 1 shows the mean annual numbers of active tuberculosis cases identified, via each component of the screening policy, between 2007 and 2014. Over this period, in terms of the mean annual number of active cases detected, contact investigation appeared to be the most successful component (mean: 28; 95% UI: 21–36), followed by the follow-up of asylum seekers (mean: 11; 95% UI: 8–16) and the screening of prisoners (mean: 10; 95% UI: 8–13). Over the same period, the supplementary initial screening of asylum seekers and the screening of other immigrants from high-incidence countries only revealed a mean of 0.38 (95% UI: 0.10–0.83) and 0.18 (95% UI: 0.02–0.51) of an active case per year, respectively, while no active tuberculosis cases were identified during screening in juvenile detention centres.

**Table 1:** Number of people screened and found positive for active tuberculosis and the associated costs, Flanders, Belgium, 2014

Screening component	No. of people		Estimated cost, in € (95% UI)
	Screened	Identified as active cases	
**Systematic screening of risk groups**	12 832	28	471 283 (452 472–490 462)
**Asylum seekers**	2 173	11	216 179 (200 572–232 554)
Follow-up after initial screening at Immigration Office	164^a^	10	56 114 (52 613–59 941)
Supplementary initial screening	195	0	36 980 (30 579–43 926)
Periodic screening	1 814	1	123 085 (111 032–135 824)
**Undocumented migrants in detention centres**	2 559	2	97 878 (85 806–110 346)
**Other immigrants from high-incidence countries**	37	0	39 555 (32 180–48 006)
**Prisoners**	7 901	15	113 325 (102 238–125 148)
**Youth in juvenile detention centres**	162	0	4 346 (2 259–7 613)
**Contact investigation**	3 872	34	395 224 (383 758–407 242)
**Screening others**	835	3	80 207 (68 506–92 833)
**Total**	**17 539**	**65**	**946 714 (928 711–964 892)**

€: Euro; UI: uncertainty interval.

^a^ In 2014, chest X-rays of 17 485 asylum seekers were taken at the Immigration Office by the Belgian Federal Agency for Asylum. The X-rays of 439 asylum seekers – of whom 164 were assigned to Flanders – were considered abnormal.

**Fig. 2:**
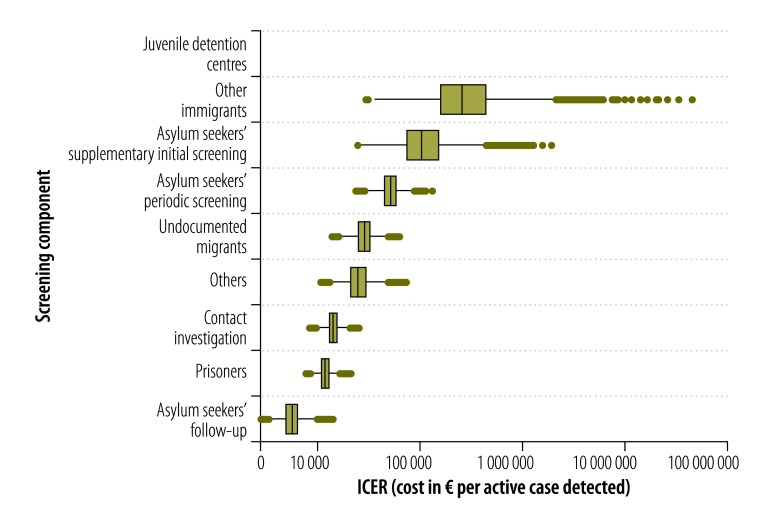
**Cost–effectiveness of each of nine components of the screening for active cases of tuberculosis, Flanders, Belgium, 2007–2014**

The screening components found to have the lowest mean costs, per active case detected (Fig. 2), were the follow-up of asylum seekers (€5564; 95% UI: 3791–8160), systematic screening of prisoners (€11 603; 95% UI: 9010–14 909) and contact investigation (€13 941; 95% UI: 10 723–18 201). The costs per case detected for the screening of others and the systematic screening of undocumented migrants in detention centres were relatively high, at €25 337 (95% UI: 15 843–40 343) and €28 474 (95% UI: 18 734–42 439), respectively. The corresponding costs for the periodic screening (€51 813; 95% UI: 34 855–76 847) and supplementary initial screening of asylum seekers (€126 236; 95% UI: 41 984–347 822) and the screening of other new immigrants from high-incidence countries (€418 359; 95% UI: 74 975–1 686 588) were even higher.

€: Euro; ICER: incremental cost–effectiveness ratio.

Notes: Others include individuals who do not belong to any specific risk groups but presented for consultation at a centre for respiratory health care. The boxplots reflect the statistical uncertainty in both the mean annual cost and the mean annual number of detected cases. In each boxplot, the vertical line represents the median of all simulated values, the box indicates the first and third quartiles, the whiskers extend to 1.5 times the interquartile range, and the points represent simulated values outside that interval. No cost–effectiveness ratio could be shown for the detection of cases during screening in juvenile detention centres because between 2007 and 2014 no active cases were detected during such screening.

**Fig. 3:**
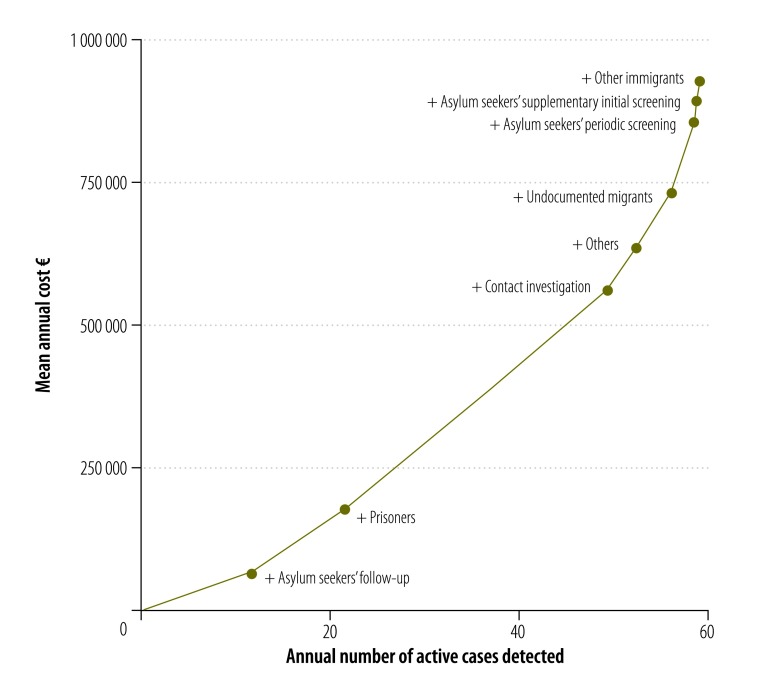
**Mean annual costs for – and annual numbers of cases identified through – increasingly complex combinations of the components of screening for active cases of tuberculosis, Flanders, Belgium, 2007–2014**

In Fig. 3, we plotted costs on the vertical axis and the number of identified active tuberculosis cases on the horizontal axis. The origin represents doing nothing – i.e. no active screening. The line connects the origin with the policy component with the lowest cost per case detected; as it runs to the right, each new data point represents the addition of another screening component and the components are added in ascending order of the incremental cost–effectiveness ratio.

€: Euro.

Note: Others include individuals who do not belong to any specific risk groups but presented for consultation at a centre for respiratory health care.

In Flanders, a focus on the screening of asylum seekers – using a combination of follow-up, supplementary initial screening and periodic screening – would have cost about €15 729 (95% UI: 11 402–21 530) per active case detected. Our estimates of the cost–effectiveness of various other combinations of the screening components are available from the corresponding author.

## Discussion

For the control of tuberculosis, WHO stresses the importance of prioritizing key interventions and target groups – based on epidemiological and health-care system assessments and an evaluation of the benefits, risks and costs of each potentially useful intervention in each setting.^6^^,^^19^ Poorly targeted systematic screening can be very expensive and ineffective^20^ and has little epidemiological impact.^21^ Screening for active tuberculosis should therefore be carefully targeted at groups with the highest tuberculosis risk, and any control programmes should incorporate measurements of effectiveness.^19^

Evaluations of the cost–effectiveness of a complete tuberculosis screening policy – in terms of the costs per active case detected – are rare. An ongoing study to evaluate and optimize tuberculosis control in the Netherlands includes an assessment of the cost–effectiveness of each of the control programme’s components.^22^^,^^23^ Other related investigations have tended to focus on the cost–effectiveness of one specific screening component and/or the detection methods used.^24^^–^^27^ One review indicated that screening of migrants as they entered a low-incidence country was not very cost-effective and had little impact on overall tuberculosis trends.^25^ In the Netherlands, the use of X-rays to screen immigrants on entry has been shown to be cost-effective only for immigrants from areas where tuberculosis incidence exceeds 300 cases per 100 000 population.^23^^,^^28^ As such screening appears to have little impact on the general epidemiology of tuberculosis in the Netherlands, the detection of latent tuberculosis infection in immigrant risk groups and the provision of accessible preventive treatment may represent better uses of the resources available.^29^ In the United Kingdom of Great Britain and Northern Ireland, the post-entry screening of immigrants for latent infection appeared to be cost-effective when the immigrants targeted came from countries with high incidence of tuberculosis.^26^ Little published information exists on the cost–effectiveness of screening individuals who are very hard to reach – e.g. homeless people^30^^,^^31^ – or on the screening of prisoners.

In Belgium, activities to detect, prevent and/or treat tuberculosis are carried out by many different institutions, with many complex interactions. This study was conducted from the perspective of the Flemish Agency for Care and Health and the Flemish Association for Respiratory Health and Tuberculosis Control – i.e. the institutions that, together, are responsible for all of the tuberculosis screening activities funded by the Flemish government. As it was not conducted from a broad societal perspective, we will have underestimated the total cost of tuberculosis detection and prevention in Flanders.

According to our estimates, in Flanders, the follow-up of asylum seekers represented the most cost-effective component of the screening for active tuberculosis. However, asylum seekers are only followed-up when they have been both found to have an abnormal chest X-ray and assigned to Flanders. As people with abnormal chest X-rays are relatively likely to have pulmonary tuberculosis, it is not surprising that a relatively high number of active cases were detected within the small group of asylum seekers who were followed up.

Contact investigation is an essential component of tuberculosis control and elimination strategies in most low-incidence countries.^32^^,^^33^ In Flanders, 34 (0.9%) and 556 (14%) of the 3872 contact investigations in 2014 resulted in the diagnosis of active tuberculosis and latent tuberculosis infection, respectively (GSA Smit, personal communication, 2015). The International Union Against Tuberculosis and Lung Disease has encouraged the prioritization of tuberculosis prevention and control in penitentiary settings.^34^ Our estimates of the cost–effectiveness of the screening of prisoners were probably too low as they ignored the costs of X-ray screening by the Justice Federal Public Service.

Among the screening activities for asylum seekers, the supplementary initial screening was found to be the least cost-effective, followed by periodic screening. The costs of these two components, per active case detected, were very high – and only exceeded by the costs of screening other immigrants – mainly because these activities revealed very few active cases. Indeed, such screening is complicated by its voluntary nature and the difficulties in reaching targeted individuals.

The screening of other new immigrants from high-incidence countries was found to be a particularly expensive method of identifying active cases of tuberculosis. Although there have been efforts to increase the number of such immigrants screened in Flanders – via networking, collaboration with relevant intermediary organizations and the use of invitations and leaflets – the annual number of such immigrants who present for screening has been declining.^35^^,^^36^ It is therefore fortunate that, according to data for 2013 from the Flemish Association for Respiratory Health and Tuberculosis Control, such immigrants are at relatively low risk of tuberculosis. If this epidemiological assessment changes, additional and more efficient communication channels may become necessary.^37^

Finally, since no active tuberculosis cases were detected in the juvenile detention centres between 2007 and 2014, we were not able to calculate the incremental cost–effectiveness ratio for this component. Although the cost of taking a mobile X-ray unit to each detention centre once a year is not particularly high – compared with the costs of the other policy components we considered – the fact that no cases were detected in the detention centres over a period of seven years makes the value of this component questionable.

Disinvestment in one or more policy components should be supported by a recent assessment of the epidemiology of tuberculosis in the setting involved. Tuberculosis incidence may be affected by changes in the influx of asylum seekers and immigrants. The number of first-time asylum seekers who applied for international protection in the European Union during the third quarter of 2015 was almost twice the number who applied in the second quarter of the same year: 413 800 versus 213 200. In Belgium this trend was even more marked, with a corresponding rise from 5040 in the second quarter of 2015 to 14 650 in the third.^38^ However, tuberculosis is not often transmitted from migrants to the resident population^39^ and WHO’s Regional Office for Europe continues to provide support in response to the migrant and refugee crisis.^40^ Possible cuts in public health spending and social protection may also permit increases in tuberculosis incidence and mortality.^9^^,^^41^

WHO has recommended that efforts to control tuberculosis in most countries be strengthened because the global incidence of the disease is only declining slowly while the threat posed by multidrug resistance is growing.^6^^,^^8^^,^^13^ However, as global incidence declines, the detection of active cases becomes increasingly rare and, in consequence, the cost–effectiveness of tuberculosis detection and prevention declines.

Our conclusions are based on the cost–effectiveness of the current implementation of each main component of the Flemish policy on tuberculosis screening. However, the cost–effectiveness of each main component can be altered by changes in each aspect of that component. If, for example, the Belgian government were to change its definition of a country with a high incidence of tuberculosis – e.g. by doubling the threshold to more than 100 cases per 100 000 – the cost–effectiveness of screening immigrants from high-incidence countries is likely to change markedly.

It may be beneficial to investigate the cost–effectiveness of screening for latent tuberculosis infection and the effectiveness and cost–effectiveness of the initial screening of all asylum seekers – regardless of their country of origin – by the Federal Agency for Asylum. In the control of tuberculosis, some countries of origin of immigrants appear to be particularly challenging – e.g. the Syrian Arab Republic has a low reported incidence of tuberculosis but mass migration and war may have substantially increased incidence since the last nationwide survey.^16^

Our study was limited by the consideration of contact investigation as an independent component in our calculations. In reality, contact investigation is unlikely to be fully independent of other screening components. To make more accurate estimates of cost–effectiveness of contact investigation, we need information on how the infective cases – i.e. the sources – are identified, the type of risk group they represent – if any – and how many contacts become infected per source.

Although health-care contexts and systems in many countries are not directly comparable, the methods used in this study could be applied in most low-incidence countries – where the epidemiology and challenges are generally similar to those in Flanders. Compared with passive detection, current programmes of active screening in low-incidence countries only detect relatively small numbers of active tuberculosis cases – and often at a high cost. The small numbers of active cases detected by active screening may reflect low numbers of active tuberculosis cases in the study population and/or inefficient screening procedures. Implementation of different procedures that are cheaper and increase coverage and/or detection rates will increase efficiency.

In Belgium, we recommend continuing contact investigation, the systematic screening of prisoners and the follow-up of asylum seekers with abnormal chest X-rays. Due to their relatively high costs, per active tuberculosis case identified, we also recommend the re-evaluation of screening in juvenile detention centres, the screening of new immigrants and the periodic and supplementary initial screening of asylum seekers.

## References

[1] Global Tuberculosis Report 2015. Geneva: World Health Organization; 2015. Available from: http://apps.who.int/iris/bitstream/10665/191102/1/9789241565059_eng.pdf [cited 2016 Oct 20].

[2] Global strategy and targets for tuberculosis prevention, care and control after 2015. Sixty-seventh World Health Assembly Resolution A67/11. Geneva: World Health Organization; 2014.

[3] Lönnroth K, Jaramillo E, Williams BG, Dye C, Raviglione M. Drivers of tuberculosis epidemics: the role of risk factors and social determinants. Soc Sci Med. 2009 6;68(12):2240–6. 10.1016/j.socscimed.2009.03.041 19394122

[4] D’Ambrosio L, Dara M, Tadolini M, Centis R, Sotgiu G, van der Werf MJ, et al.; European national programme representatives. Tuberculosis elimination: theory and practice in Europe. Eur Respir J. 2014 5;43(5):1410–20. 10.1183/09031936.00198813 24389868

[5] Dara M, de Colombani P, Petrova-Benedict R, Centis R, Zellweger JP, Sandgren A, et al.; Wolfheze Transborder Migration Task Force. Minimum package for cross-border TB control and care in the WHO European region: a Wolfheze consensus statement. Eur Respir J. 2012 11;40(5):1081–90. 10.1183/09031936.00053012 22653772 PMC3485571

[6] Framework towards tuberculosis elimination in low-incidence countries. Geneva: World Health Organization; 2014. 25473715

[7] Creswell J, Raviglione M, Ottmani S, Migliori GB, Uplekar M, Blanc L, et al. Tuberculosis and noncommunicable diseases: neglected links and missed opportunities. Eur Respir J. 2011 5;37(5):1269–82. 10.1183/09031936.00084310 20947679

[8] Nathanson E, Nunn P, Uplekar M, Floyd K, Jaramillo E, Lönnroth K, et al. MDR tuberculosis – critical steps for prevention and control. N Engl J Med. 2010 9 9;363(11):1050–8. 10.1056/NEJMra0908076 20825317

[9] Reeves A, Basu S, McKee M, Sandgren A, Stuckler D, Semenza JC. Tuberculosis control and economic recession: longitudinal study of data from 21 European countries, 1991–2012. Bull World Health Organ. 2015 6 1;93(6):369–79. 10.2471/BLT.14.142356 26240458 PMC4450704

[10] Floyd K, Pantoja A. Financial resources required for tuberculosis control to achieve global targets set for 2015. Bull World Health Organ. 2008 7;86(7):568–76. 10.2471/BLT.07.049767 18670669 PMC2647490

[11] Verdier JE, de Vlas SJ, Baltussen R, Richardus JH. A systematic review of economic evaluation studies of tuberculosis control in high-income countries. Int J Tuberc Lung Dis. 2011 12;15(12):1587–98. 10.5588/ijtld.10.0332 21740647

[12] Tuberculosis register. Brussels: Flemish Association for Respiratory Health and Tuberculosis Control; 2014 Dutch.

[13] Global tuberculosis database [Internet]. Geneva: World Health Organization. Available from: http://who.int/tb/country/data/download/en/[cited 2015 Jul 7].

[14] Husereau D, Drummond M, Petrou S, Carswell C, Moher D, Greenberg D, et al.; CHEERS Task Force. Consolidated Health Economic Evaluation Reporting Standards (CHEERS) statement. Value Health. 2013 Mar-Apr;16(2):e1–5. 10.1016/j.jval.2013.02.010 23565948

[15] Population by place of residence, nationality, marital status, age and sex – 1 January 2015. Brussels: Federal Public Service Economy; 2015. Available from: https://bestat.economie.fgov.be/bestat/crosstable.xhtml?datasource=65ee413b-3859-4c6f-a847-09b631766fa7 [cited 2016 Jan 6]. Dutch.

[16] Arrazola de Onate W, Weber L, Janssens K, Wanlin M, Forier A, Schol S, et al. Tuberculosis screening yield of asylum seekers in Europe – correspondence. Eur Respir J. 2016;48(4):1–2. 10.1183/13993003.00991-2016 27694419

[17] Bilcke J, Beutels P, Brisson M, Jit M. Accounting for methodological, structural, and parameter uncertainty in decision-analytic models: a practical guide. Med Decis Making. 2011 Jul-Aug;31(4):675–92. 10.1177/0272989X11409240 21653805

[18] R: a language and environment for statistical computing. Vienna: R Core Team, R Foundation for Statistical Computing; 2015. Available from: http://www.R-project.org/ [cited 2016 Jan 6].

[19] Systematic screening for active tuberculosis – principles and recommendations. Geneva: World Health Organization; 2013. 25996015

[20] Van’t Hoog AH, Onozaki I, Lönnroth K. Choosing algorithms for TB screening: a modelling study to compare yield, predictive value and diagnostic burden. BMC Infect Dis. 2014;14(1):532. 10.1186/1471-2334-14-532 25326816 PMC4287425

[21] Kranzer K, Afnan-Holmes H, Tomlin K, Golub JE, Shapiro AE, Schaap A, et al. The benefits to communities and individuals of screening for active tuberculosis disease: a systematic review. Int J Tuberc Lung Dis. 2013 4;17(4):432–46. 10.5588/ijtld.12.0743 23485377

[22] de Vries G, Baltussen R. The cost of tuberculosis and tuberculosis control. Infectieziekten Bull. 2013;24:136–40. Dutch.

[23] Report of the joint WHO and ECDC programme review of the national TB control programme in the Netherlands 2013. Copenhagen: World Health Organization Regional Office for Europe; 2014. Available from: http://www.euro.who.int/__data/assets/pdf_file/0006/255354/Report-of-the-joint-WHO-and-ECDC-programme-review-of-the-national-TB-control-programme-in-the-Netherlands.pdf[cited 2016 Feb 8].

[24] Erkens CG, Dinmohamed AG, Kamphorst M, Toumanian S, van Nispen-Dobrescu R, Alink M, et al. Added value of interferon-gamma release assays in screening for tuberculous infection in the Netherlands. Int J Tuberc Lung Dis. 2014 4;18(4):413–20. 10.5588/ijtld.13.0589 24670695

[25] Dasgupta K, Menzies D. Cost–effectiveness of tuberculosis control strategies among immigrants and refugees. Eur Respir J. 2005 6;25(6):1107–16. 10.1183/09031936.05.00074004 15929967

[26] Pareek M, Watson JP, Ormerod LP, Kon OM, Woltmann G, White PJ, et al. Screening of immigrants in the UK for imported latent tuberculosis: a multicentre cohort study and cost–effectiveness analysis. Lancet Infect Dis. 2011 6;11(6):435–44. 10.1016/S1473-3099(11)70069-X 21514236 PMC3108102

[27] Zenner D, Southern J, van Hest R, DeVries G, Stagg HR, Antoine D, et al. Active case finding for tuberculosis among high-risk groups in low-incidence countries. Int J Tuberc Lung Dis. 2013 5;17(5):573–82. 10.5588/ijtld.12.0920 23575321

[28] Van Rest JF, Erkens CGM, de Vries G. Evaluation of the tuberculosis screening of immigrants in the Netherlands, 2005-2010. The Hague: KNCV Tuberculosis Foundation; 2012. Available from: https://www.kncvtbc.org/uploaded/2016/01/evaluatie_tuberculosescreening_immigranten_2005-2010.pdf [cited 2016 Feb 8]. Dutch.

[29] Erkens C, Verhagen M. Discussion about TB screening of immigrants. Tegen Tuberc. 2014;110(2):10–1. Dutch.

[30] Jit M, Stagg HR, Aldridge RW, White PJ, Abubakar I; Find and Treat Evaluation Team. Dedicated outreach service for hard to reach patients with tuberculosis in London: observational study and economic evaluation. BMJ. 2011;343 sep13 5:d5376–5376. 10.1136/bmj.d5376 22067473 PMC3273731

[31] Kimerling ME, Shakes CF, Carlisle R, Lok KH, Benjamin WH, Dunlap NE. Spot sputum screening: evaluation of an intervention in two homeless shelters. Int J Tuberc Lung Dis. 1999 7;3(7):613–9. 10423224

[32] Recommendations for investigating the contacts of persons with infectious tuberculosis in low- and middle-income countries. Geneva: World Health Organization; 2012. 24404639

[33] Erkens CGM, Kamphorst M, Abubakar I, Bothamley GH, Chemtob D, Haas W, et al. Tuberculosis contact investigation in low prevalence countries: a European consensus. Eur Respir J. 2010 10;36(4):925–49. 10.1183/09031936.00201609 20889463

[34] Dara M, Chadha SS, Vinkeles Melchers NV, van den Hombergh J, Gurbanova E, Al-Darraji H, et al.; International Union Against Tuberculosis and Lung Disease. Time to act to prevent and control tuberculosis among inmates. Int J Tuberc Lung Dis. 2013 1;17(1):4–5. 23231999 10.5588/ijtld.12.0909

[35] Annual report on tuberculosis detection and control in the Flemish region. Brussels: Flemish Association for Respiratory Health and Tuberculosis Control; 2013. Dutch.

[36] Annual report on tuberculosis detection and control in the Flemish region. Brussels: Flemish Association for Respiratory Health and Tuberculosis Control; 2014. Dutch.

[37] Tuberculosis register. Brussels: Flemish Association for Respiratory Health and Tuberculosis Control; 2013. Available from: http://www.vrgt.be/uploads/documentenbank/28bf5af1188e3e5846bbce9db29bf728.pdf [cited 2016 Jan 6]. Dutch.

[38] Asylum in the EU Member States. More than 410 000 first time asylum seekers registered in the third quarter of 2015. 1 out of 3 are from Syria. Luxembourg: Eurostat; 2015. Available from: http://ec.europa.eu/eurostat/documents/2995521/7105334/3-10122015-ap-en.pdf/04886524-58f2-40e9-995d-d97520e62a0e [cited 2016 Jan 4].

[39] Kamper-Jørgensen Z, Andersen AB, Kok-Jensen A, Kamper-Jørgensen M, Bygbjerg IC, Andersen PH, et al. Migrant tuberculosis: the extent of transmission in a low burden country. BMC Infect Dis. 2012;12(1):60. 10.1186/1471-2334-12-60 22423983 PMC3342118

[40] Migration and health: key issues [Internet]. Geneva: World Health Organization; 2015. Available from: http://www.euro.who.int/en/health-topics/health-determinants/migration-and-health/news/news/2015/09/population-movement-is-a-challenge-for-refugees-and-migrants-as-well-as-for-the-receiving-population/migration-and-health-key-issues[cited 2016 Jan 4].

[41] Reeves A, Basu S, McKee M, Stuckler D, Sandgren A, Semenza J. Social protection and tuberculosis control in 21 European countries, 1995–2012: a cross-national statistical modelling analysis. Lancet Infect Dis. 2014 11;14(11):1105–12. 10.1016/S1473-3099(14)70927-2 25303845

